# Criterion validity of the activPAL^TM^ and ActiGraph for assessing children’s sitting and standing time in a school classroom setting

**DOI:** 10.1186/s12966-016-0402-x

**Published:** 2016-07-07

**Authors:** Kate Ridley, Nicola D. Ridgers, Jo Salmon

**Affiliations:** Sport, Health and Physical Education (SHAPE) Research Centre, School of Education, Flinders University, Adelaide, SA Australia; Institute for Physical Activity and Nutrition (IPAN), School of Exercise and Nutrition Sciences, Deakin University, Geelong, Australia

**Keywords:** Accelerometry, Inclinometry, Youth, Sedentary

## Abstract

**Background:**

Few studies have investigated the accuracy of the ActiGraph (AG) GTX3 accelerometer for assessing children’s sitting and standing time. The activPAL (aP) has an inclinometer function that enables it to distinguish between sitting/lying and standing; however, its accuracy for assessing sitting and standing in older children is unknown. This study validated the accuracy of these devices for estimating sitting and standing time in a school classroom against a criterion measure of direct observation (DO).

**Findings:**

Forty children in grades 5–7 wore both devices while being video recorded during two school lessons. AG and aP data were simultaneously collected in 15-s epochs. Individual participant DO and aP data were recorded as total time spent sitting/lying, standing and stepping. AG data were converted into time spent sitting and standing using previously established cut-points. Compared with DO, the aP underestimated sitting time (mean bias = -1.9 min, 95 % LoA = -8.9 to 5.2 min) and overestimated standing time (mean bias = 1.8 min, 95 % LoA = -9.6 to 13.3 min). The best-performing AG cut-point across both sitting and standing (<75 counts/15 s) was more accurate than the aP, underestimating sitting time (mean bias = -0.8 min, 95 % LoA = -10.5 to 9.9 min) and standing time (mean bias = -0.4 min, 95 % LoA = -9.8 to 9.1 min), but was less precise as evidenced by wider LoAs and poorer correlations with DO (sitting r = 0.86 aP vs 0.80 AG; standing r = 0.78 aP vs 0.60 AG).

**Conclusions:**

The aP demonstrated good accuracy and precision for assessing free-living sitting and standing time in classroom settings. The AG was most accurate using a cut-point of < 75 counts/15 s. Further studies should validate the monitors in settings with greater inter- and intra-individual variation in movement patterns.

## Background

There is mixed evidence of associations between objectively measured volume or patterns of sedentary behaviour and cardiometabolic, psychosocial health and academic outcomes [1–3]. Sedentary behaviour, the time children spend sitting during waking hours while expending ≤1.5 METs [4], must be measured accurately and distinguished from standing to better understand associations with health. The combination of an energy expenditure threshold and a postural allocation in the sedentary definition complicates measurement.

Accelerometry has typically been used to estimate total physical activity or time spent in moderate- to-vigorous-intensity physical activity, with the hip-worn ActiGraph (AG; Pensacola, FL, USA) commonly used in research with children [5]. This device is not able to directly assess sitting time; however, it is used to determine time spent in sedentary behaviour based on a lack of movement under a specified counts-per-time-period threshold. This threshold (or cut-point) is based on comparisons with criterion measures of energy expenditure (EE) measured by indirect calorimetry and derived from regression equations or receiver operating curve (ROC) analyses [6, 7]. Using accelerometer cut-points to determine sedentary time is therefore most closely aligned to the EE component of the definition of sedentary behaviour, yet the lack of a postural measurement leads to a risk of misclassifying standing still as sitting [7].

More recently, inclinometers have been used as an alternate objective measure of sedentary behaviour [7, 8]. Rather than classifying movements via acceleration counts, the angle and position of the thigh-mounted inclinometer are detected, and the position is classified as either sitting/lying, standing or stepping. Therefore, inclinometry allows researchers to distinguish between postures and directly assess sitting time [8]. The activPAL™ [aP; PAL Technologies Ltd., Glasgow, UK] is a commonly used inclinometer in physical activity and sedentary behaviour research studies [7, 9].

Studies have assessed the concurrent agreement between the aP and AG [7] or the validity of the aP compared to a criterion measure of observation [8, 9] showing good agreement. However, few studies have investigated the criterion validity of the monitors for both sitting and standing using an observational design among older primary school children. Therefore, this study builds on our work published in 2012 [7] by assessing the validity of the aP and AG to accurately detect sitting while children perform typical activities in a school classroom compared to the criterion measure of direct observation. Secondary aims were to: a) assess the validity of the monitors to accurately measure standing compared to direct observation; b) compare the validity of the commonly cited sedentary cut-points for both detecting sitting and distinguishing between sitting and standing; and c) to assess the aP’s ability to detect transitions between sitting and upright (standing) postures.

## Methods

### Participants and procedure

A convenience sample was recruited from one school in suburban Adelaide, Australia. The non-government school has an Index of Community Socio-Educational Advantage (ISCEA) score higher than the national average (i.e. 1138 vs 1000). The School Principal and parents/care givers provided written consent. All children in three separate classes (*n* = 65, grades 5, 6 & 7, ages 9-12 y) were invited to participate with 48 consenting (response rate = 74 %). The Flinders Clinical Research Ethics Committee granted approval for this study.

Teachers nominated two classroom lessons for observation, one involving a high prevalence of sitting, and another where more movement around the classroom was permitted (total *n* = 6 lessons across the 3 grade levels). Children undertook their usual classroom lessons (i.e. language, science and geography) with no researcher intervention. Researchers fitted participants with aP inclinometers enclosed in a small pocket within an adjustable elasticised belt and secured at the mid-anterior position on the thigh [7]. AG (GT3X model) accelerometers were hip mounted on an adjustable belt. Both the aP and AG collected data in 15 s epochs. Three stationary tripod mounted video cameras recorded the entire classroom during lessons. The aP, AG and video footage were synchronised for start and stop times.

### Measures and data treatment

Data were downloaded using aP (v5.8.3.5) and AG (v5.10.0) software. aP data were summarised into time spent sitting/lying, standing and stepping. AG data were converted into minutes spent sedentary via MeterPlus software (v4.2, Santech) using the following cut-points: < 13, < 25, < 38 and < 75 counts per 15 s, corresponding to the commonly cited per-minute cut-points of 50, 100, 150 and 300 counts · min^−1^ [5, 7]. AG data were converted into time spent standing by using the aforementioned cut-points as lower limits and < 200 counts per 15 s as the upper limit. This upper limit corresponds to the 800 counts · min^−1^ cut-point previously used to classify ‘low-light intensity physical activity’ comprising static light activities such as standing [10].

Video footage from the three cameras were imported into Dartfish Team Pro tagging software (Dartfish, Fribourg, Switzerland) and merged into one synchronised file. The video file was subsequently reviewed by a trained researcher and continuous direct observation (DO) data collected using SOFIT data collection protocols for assessing activity level (lying down, sitting, standing, walking or vigorous activity) [11]. The walking and vigorous categories were combined for this study. There were no instances of lying down. The video files were reviewed separately for each participant and lesson, resulting in 80 observation files. Files were viewed at half-speed resulting in approximately 90 h of coding. Eleven per cent of these files were double coded by a second trained researcher. The inter-rater reliability for sitting time was excellent (ICC = 0.99).

### Statistical analyses

Sitting time data were normally distributed across all three measures. Standing time data were skewed so medians will be used for descriptive purposes. Bias (monitor derived time minus directly observed time) data were normally distributed for both sitting and standing. The accuracy of the aP and AG cut-points were assessed by calculating mean bias in minutes. The ability of the monitors to accurately estimate group means was calculated using a percentage bias where the mean monitored sitting time of the entire sample was divided by the mean observed sitting time of the entire sample. Due to the skewed nature of the data the percentage bias calculation was not performed for the standing data. Precision (i.e. amount of random error present) was evaluated with 95 % limits of agreement (LoA) calculated using the Bland-Altman method for accounting for two observations per individual, and Spearman correlations. Paired t-tests compared the number of postural transitions identified by the aP compared to DO.

## Results

**Fig. 1:**
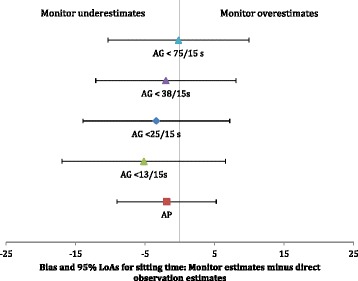
Illustration of the bias (filled shapes) and 95 % limits of agreement (LoAs) adjusted for two observations for individual (horizontal error bars) for the activPAL and selected ActiGraph cut-points for sitting. Note: aP = activPAL; AG < 13/15 s = ActiGraph cut point of <13 counts/15 s; AG < 25/15 s = ActiGraph cut point of <25 counts/15 s; AG < 38/15 s = ActiGraph cut point of <38 counts/15 s; AG < 75/15 s = ActiGraph cut point of <75 counts/15 s

**Fig. 2:**
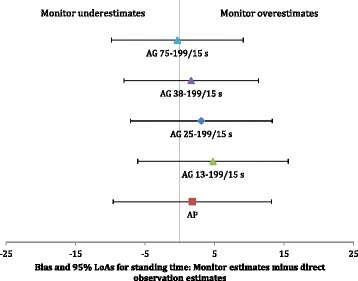
Illustration of the bias (filled shapes) and 95 % limits of agreement (LoAs) adjusted for two observations for individual (horizontal error bars) for the activPAL and selected ActiGraph cut-point ranges for standing. Note: aP = activPAL; AG 75-199/15 s = ActiGraph cut point range 75-199 counts/15 s; AG 38-199/15 s = ActiGraph cut point range 38-199 counts/15 s; AG 25-199/15 s = ActiGraph cut point range 25-199 counts/15 s; AG 13-199/15 s = ActiGraph cut point range 13-199 counts/15 s

Forty children (53 % boys) provided complete data for two lessons, resulting in *n* = 80 observations. Eight children were excluded due to being absent for one of the observed lessons or having left the classroom during the observation period. The mean (± SD) observed lesson time was 36.3 ± 6.1 min. Across all lessons, the mean (SD) directly observed sitting time was 33.5 (7.6) min, the median (IQR) standing time was 0.8 (2.3) min, and the mean (SD) number of postural transitions was 8 (8). Figures 1 and 2 show the mean bias and 95 % LoAs for sitting and standing time in minutes across monitors and AG cut-points. The aP demonstrated good accuracy for sitting with a mean bias at the individual level of -1.9 min (95 % LoA = -9.0 to 5.2 min), resulting in a 5.6 % underestimation at the group level. The AG cut-point of <75 counts/15 s (<300 counts · min^−1^) demonstrated smaller mean biases (mean bias = -0.2 min, 95 % LoA = -10.5 to 9.9 min), resulting in a 2.4 % underestimation of sitting at the group level; however, wide LoAs (-10.5 to 9.9 min) indicate poor precision. The aP overestimated standing by +1.8 min (95 % LoA = -9.6 to 13.3 min), while the AG 75-200 counts/15 s range slightly underestimated standing with a mean bias = -0.4 min, again with wide LoAs (95 % LoA = -9.8 to 9.1 min).

The poorer precision of the AG is also reflected in weaker correlations with DO across both sitting and standing (see Table 1). There were no significant differences in the number of postural transitions identified by the aP compared to DO (mean diff = 0.24, *p* = 0.44).

**Table 1:** Spearman Correlations (Rho) between monitor-determined and directly observed sitting and standing time

Monitor	Correlation (*Rho*) with DO	
	Sitting	Standing#
aP	0.86^**^	0.78^**^
AG < 13 counts/15 s (50 cpm)	0.72^**^	0.62^**^
AG < 25 counts/15 s (100 cpm)	0.76^**^	0.61^**^
AG < 38 counts/15 s (150 cpm)	0.79^**^	0.63^**^
AG < 75 counts/15 s (300 cpm)	0.80^**^	0.60^**^

*Note:* ^**^ = all Spearman correlations were statistically significant at *p* < 0.01; ^#^Standing values for AG calculated using a count range with 200 counts/15 s (800 cpm) as the upper end cut-point and the designated cut-point as the lower end cut-point

*DO* direct observation, *aP* activPal, *AG* ActiGraph.

## Discussion and conclusions

Both devices demonstrated good accuracy for measuring group estimates of sitting time in the natural classroom setting. We observed behaviour for the duration of one lesson, yet if we apply the mean percentage bias to an assumed 330 min of classroom time per day (i.e. excluding break times), we predict the group mean underestimation for sitting across a school day would be approximately 18.5 min for the aP and approximately 8 min per day for the AG using a cut point of 75 counts/15 s. However, precision was influenced by large errors in some individual observations, resulting in wide LoAs, particularly for the AG. Poor precision has implications for statistical power and the ability to detect both associations with health outcomes and intervention induced changes in behaviour. Where large errors occurred, the DO data revealed the devices were affected by different types of errors. Irregular sitting styles where children sat on the edge of their chair and the thigh position was more vertical than a normal seated position hampered the ability of the aP to accurately classify sitting. In contrast, DO data at the individual level revealed the AG overestimated sedentary time when students stood very still for the majority of the lesson, resulting in low accelerometry counts below the sedentary cut-point thresholds.

Our ability to assess the monitors’ ability to measure standing was hampered by a lack of prevalence and variability in standing time. However, our results showed both monitors significantly correlated with directly observed standing. The ability of the aP to accurately detect transitions from sit to stand/step is noteworthy due to recent evidence that interrupting sedentary behaviour in 7–11 year olds leads to improved short-term metabolic function [2].

Our previous research [7] found the 100 counts · min^−1^ cut-point using the AG had the lowest mean bias for overall daily sitting time across an entire school day compared to the aP. In contrast, the present study found <300 counts · min^−1^ to have the smallest mean bias for both sitting and standing, but poor precision. The sedentary nature of the sample is likely to have had an impact on the mean differences observed across the AG cut-points and in comparison to other studies. In this study, little time was spent in non-sedentary behaviour and few transitions occurred during the lessons; therefore, a higher cut-point was less likely to misclassify accelerations caused by fidgeting. The 75 counts/15 s may not be as accurate a measure of sitting in a more active sample with more changes in posture [7]. Nevertheless, school classrooms are sedentary in nature and our observed sitting time (92 %) was similar to that reported in another classroom-based study (97 %) [12].

This study provides evidence supporting the aP as a valid measure of sitting time, standing time, and postural transitions in children within classroom settings which are increasingly the focus of school-based interventions [13]. Our study provides some supporting evidence toward a 75 counts/15 s upper cut-point for measuring sitting time and a range of 75–200 counts/15 s (300–800 cpm) for measuring standing time, however the nature of the activity patterns in the present sample are likely to influence the optimal sedentary cut-points determined. While it might be expected that a high upper limit for sitting will perform well when classifying activity in a largely sedentary sample, the 75 counts/15 s cut-point also performed well as the lower limit for standing in this sample. The ability of monitors to distinguish between sitting and standing statically is important in order to elucidate the health risks associated with inactivity vs sedentariness. Therefore further research in more diverse settings with larger variability in activity/sedentary levels including the manipulation of both the upper and lower limits to determine a standing cut-point is recommended.

## Abbreviations

AG, ActiGraph GTX3; aP, activPAL; DO, direct observation; EE, energy expenditure; ICC, intra-class coefficient; LoA, limits of agreement; MET, metabolic equivalent; ROC, receiver operating curve
